# Primary glomangioma of the chest wall: case report and review of literature

**DOI:** 10.1093/jscr/rjaf300

**Published:** 2025-05-12

**Authors:** Zymantas Jagelavicius, Eitvile Mickeviciute, Edvardas Zurauskas, Ricardas Janilionis

**Affiliations:** Clinic of Chest Disease, Immunology and Allergology, Institute of Biomedical Sciences, Faculty of Medicine, Vilnius University, Santariskiu 2, LT-08406, Vilnius, Lithuania; Faculty of Medicine, Vilnius University, M. K. Ciurlionio 21, LT-03101, Vilnius, Lithuania; Department of Pathology and Forensic Medicine, Institute of Biomedical Sciences, Faculty of Medicine, Vilnius University, P. Baublio 5, LT-08406, Vilnius, Lithuania; Clinic of Chest Disease, Immunology and Allergology, Institute of Biomedical Sciences, Faculty of Medicine, Vilnius University, Santariskiu 2, LT-08406, Vilnius, Lithuania

**Keywords:** chest wall tumor, glomus tumor, glomangioma, video-assisted thoracic surgery, thoracic surgery

## Abstract

Glomus tumors are rare neoplasms that usually appear in subungual locations. A primary glomus tumor in the chest wall is extremely rare. We present a case of a 42-year-old male with intermittent pain under the right scapula for approximately a year. No skin lesions were observed. A magnetic resonance imaging and computed tomography scan showed a well-defined subpleural lesion on the right side of the chest along the ninth intercostal space. The tumor was removed via video-assisted thoracic surgery. The pathological report revealed a glomangioma. We analyzed in detail existing cases of glomus tumors in the chest wall. The chest wall is a possible site of a glomus tumor, which doesn’t have any specific clinical or radiological signs. Radical surgical removal should be the treatment of choice.

## Introduction

Primary chest wall tumors occur in <2% of the population and account for ~5% of all thoracic neoplasms. Less than half of chest wall tumors are benign, and >20% are discovered incidentally on chest X-rays. The histological types of benign chest wall tumors can vary widely and include osteochondromas, chondromas, fibrous dysplasias, lipomas, lymphangiomas, hemangiomas, neurogenic tumors, and others. This variability often complicates diagnostics, sometimes necessitating a biopsy for a definitive diagnosis [1, 2].

Glomus tumors are usually benign neoplasms primarily occurring in the skin, with the subungual region being the most common site. The incidence is ~1.6% of soft tissue tumors [3]. Even though the majority of these tumors appear subungual, extradigital sites like the digestive system, respiratory system, breasts, intranasal, or sellar have been reported [4–6].

Having a tumor in the chest wall is uncommon, and the occurrence of a glomus tumor in this location is even rarer. We present a case of glomangioma in the chest wall and analyze all the glomus tumors in the chest wall we found in the literature.

## Case report

**Figure 1:**
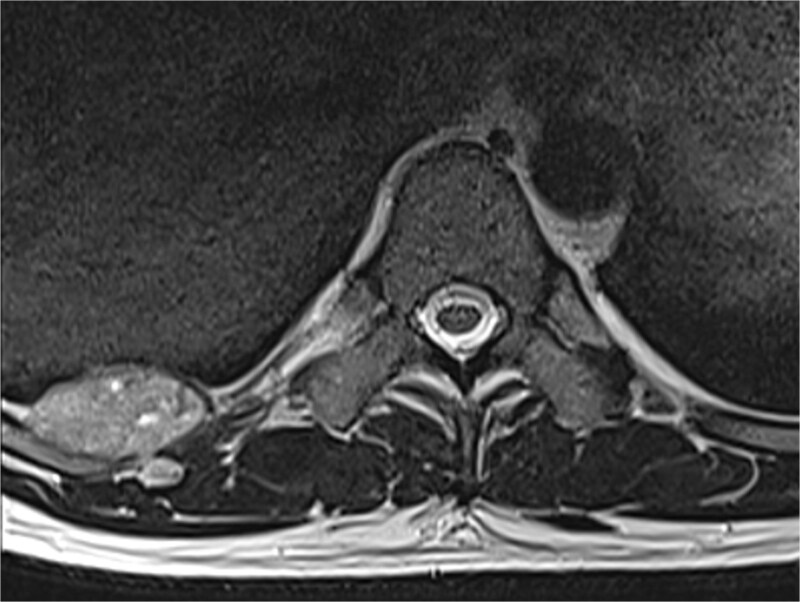
A magnetic resonance imaging scan shows a lesion on the right posterior side of the chest wall.

**Figure 2:**
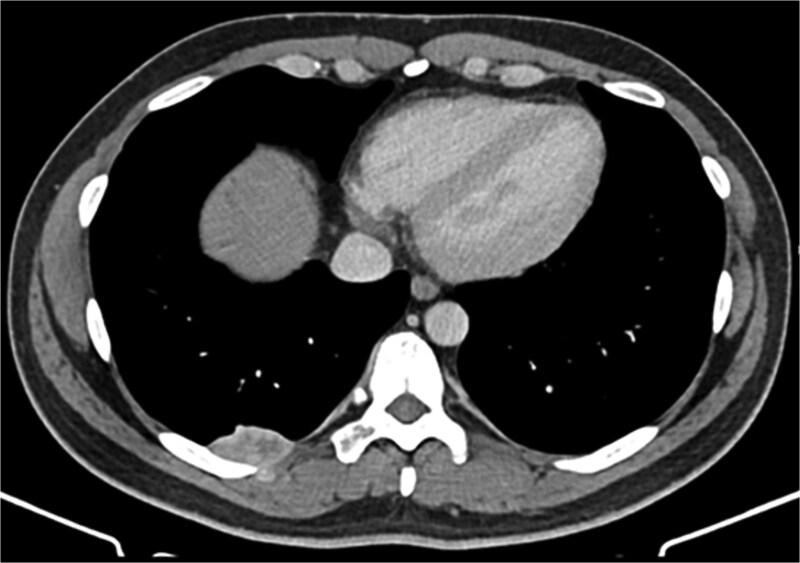
Contrast-enhanced CT scan of the chest*.*

A 42-year-old male patient presented with intermittent pain under the right scapula for one year. The pain occurred both day and night, radiated to the right side of the abdomen, and improved with exercise. No skin lesions were observed. A thoracic spine magnetic resonance imaging scan showed a well-defined subpleural lesion on the right side along the ninth intercostal space posteriorly, which was hyperintense on T1-weighted imaging and isointense on T2-weighted imaging, with contrast enhancement features. The lesion size was 38 × 18 mm and extends into the back muscles with an additional nodule of 6 × 9 mm (Fig. 1). A contrast-enhanced computed tomography (CT) scan of the chest was performed to obtain a comprehensive chest view, revealing the same lesion without any other pathological findings (Fig. 2).

**Figure 3:**
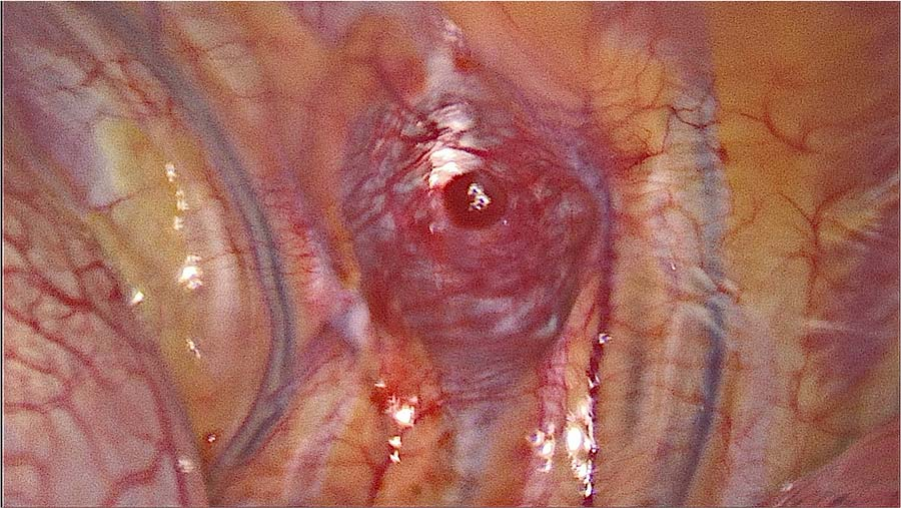
Video-assisted thoracic surgery. Intraoperative view of the tumor.

It was decided to remove the lesion using video-assisted thoracic surgery. The moderately firm mass under the parietal pleura in the ninth intercostal space was identified (Fig. 3). The tumor with the additional intramuscular portion of it was radically removed.

**Figure 4:**
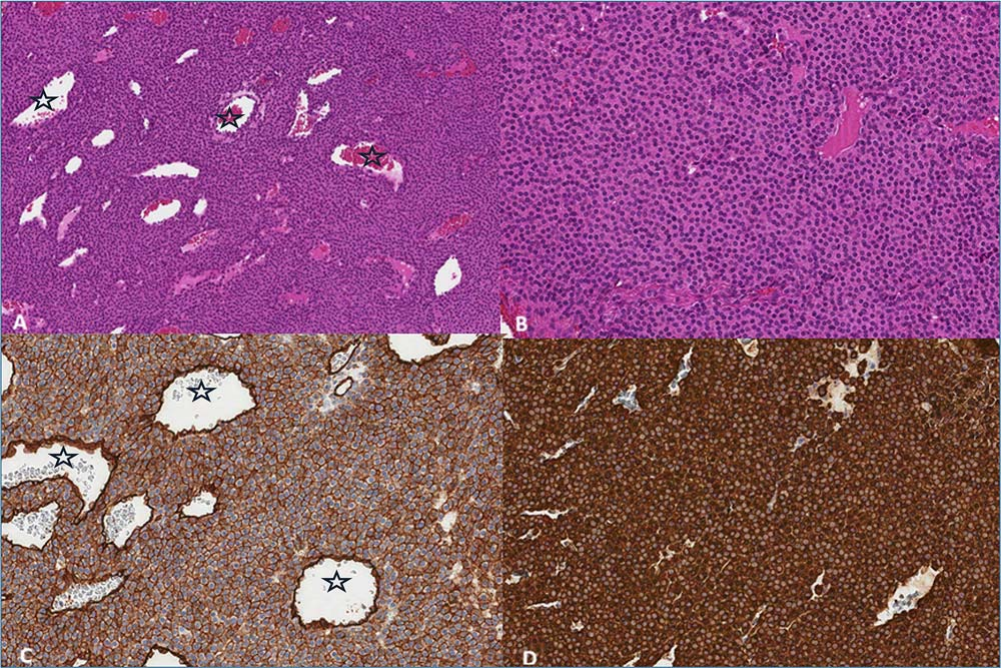
(A) Haemotoxylin & Eosin (H&E) ×100. The tumor consists of branched blood vessels (stars) lined by endothelial cells, surrounded by solid areas of uniform glomus cells. (B) H & E ×200. Glomus cells have a rounded nucleus and an eosinophilic cytoplasm. (C) CD34 ×200. Positive reaction in tumor and endothelial cells lining blood vessels (stars). (D) SMA ×200. Positive reaction in tumor cells.

The pathology report stated that the tumor fragments consist of solid areas of monomorphic cells with round nuclei exhibiting condensed chromatin and eosinophilic cytoplasm. The cell borders are well-defined, with no mitotic activity observed. The lesion contains a prominent vascular component. Immunohistochemical analysis revealed strong positivity for Caldesmon (+++), smooth muscle actin (SMA) (+++), and moderate positivity for CD34 (++) in 100% of the cells. The tumor was negative for PANCK, CD117, CD56, S-100, and desmin. The pathological diagnosis was glomangioma (Fig. 4).

The postoperative course was uneventful. On the fourth postoperative day, the patient was discharged with relief of symptoms. On the 3-month follow-up, he has no chest pain or signs of relapse. In the future, the patient will be followed by the thoracic surgeon. It is planned to perform a computed tomography scan at 6 and 12 months after the surgery, and then once a year for at least 5 years.

## Discussion

To the best of our knowledge, there are 12 cases described in the literature of glomus tumors in the chest wall. We expanded the analysis of these cases from previously published by Zanjani *et al*. [4] and later updated by Alyaseen *et al*. [7], adding some new cases and analysing them in more detail (Table 1) [4, 7–17].

**Table 1:** Comparison of cases of chest wall glomus tumors

Study Year	Gender	Age	Symptoms	Duration of symptoms	Skin involvement	Location	Size (cm)	Histological subtype	Extension to the pleura	Recurrence (follow-up time)
Schneller [8] 2001	Male	30	Pain	10 years	N.R.	Multifocal: intercostal spaces, left posterior chest wall	Up to 9	Glomangiomyoma	Yes	N.R.
Tsuruta *et al*. [9] 2003^*^	Female	19	Pain	5 years	No	Right anterior 3^rd^ intercostal space	2	Glomus	Yes	No (1 year)
Neelaiah *et al*. [10] 2005	Male	46	Pain	2 years	Bluish red nodule	Subcutaneous anterior chest wall	0.5 × 0.75	Glomus	No	N.R.
Uchiyama *et al*. [11] 2011^*^	Male	50	Pain	10 years	N.R.	Right 3rd intercostal space	N.R.	Glomus	N.R.	N.R.
Venugopal [12] 2015	Male	67	Pain, sensitivity to cold	6 years	Vague swelling	Left scapula area	2	Glomus	No	N.R.
Temiz *et al*. [13] 2016	N.R.	N.R.	Pain	N.R.	Purple-colored hard subcutaneous nodule	Sternal projection	0.5–1	Glomus	No	No (1 year)
Yim *et al*. [14] 2016	Male	41	Pain	N.R.	N.R.	Right paraspinal area	6.3	Glomus	N.R.	Yes (in 6 months, 4 years)
Kambhampati *et al*. [15] 2018	Male	47	Pain	2 years	Painful purple papule	Left anterior chest wall	1.0 × 0.4	Glomus	No	No (6 months)
Zanjani *et al*. [4] 2021	Male	63	Pain, sensitivity to cold, limited shoulder movements	15 years	Axillary lump	Left lateral chest wall	5	Glomangioma	No	No (19 months)
Alyaseen *et al*. [7] 2021	Male	35	Pain, sensitivity to cold	7 years	Bluish papule	Right lateral chest wall	1	Glomangioma	No	N.R.
Bhalchandra Londhe *et al*. [16] 2021	Female	27	Pain	N.R.	No	Right posterior chest wall muscles	2 × 1 × 1.9	Glomus	No	No (1 year)
Engle *et al*. [17] 2024	Female	67	N.R.	6 months	Skin-colored subcutaneous nodule	Anterior chest wall Mts in the lung	N.R.	Glomus	No	N.R.
Our case 2025	Male	42	Pain	1 year	No	Right posterior chest wall, 9th intercostal space	3.8 × 1.8	Glomangioma	Yes	No (3 months)

N.R. – information is not reported; ^*^-only abstract available in English

Lee *et al*. reported that out of 152 patients with glomus tumors, 42 (27.6%) were found extradigital [3]. Extradigital glomus tumors show a male predominance, while digital tumors are more common in females. The male-to-female ratio ranges from 2.7:1 to 4.6:1 [3]. In chest wall glomus tumors, the male-to-female ratio was 4:1. The average age at diagnosis for glomus tumors was 44.5 years, with a mean symptom duration of 5.9 years before diagnosis.

Glomus tumors typically present with a classic triad of intense pain, localized tenderness, and sensitivity to cold [18]. Patients with extradigital glomus tumors usually experience significantly lower rates of pain and cold intolerance compared to those with digital tumors. Lee *et al.* reported that pain was present in 56.8% of cases, while no patients with extradigital glomus tumors had sensitivity to cold [3]. Even though the pain rate was found to be significantly lower in extradigital glomus tumors, all patients with glomus tumors in the chest wall experienced pain. Cold intolerance was reported in three patients, initially making it harder to diagnose without a full triad of symptoms. In all three cases, the tumor has involved the skin. If the tumor was in deeper layers of the chest wall, there was no tenderness or reaction to temperature.

Glomus tumors typically appear as bluish or purple solitary papules [18]. Seven patients had changes in skin appearance, and four patients were reported to have a purple or bluish-red papule. As reported by Lee *et al.,* out of 38 extradigital glomus tumors, 10 (26.3%) appeared as purple papules [3]. Glomus tumors in the chest wall, presenting as skin discoloration, were more superficial and smaller (up to 1 cm) than those without color change. Most tumors with deeper locations in the chest wall were bigger (2–19 cm).

The histological classification of glomus tumors may vary depending on their morphological features: solid glomus tumor, glomangioma, and glomangiomyoma [19]. Lee *et al*. reported that most glomus tumors were solid glomus tumors [3]. Most of the tumors found in the chest wall were also solid glomus tumors, with nine patients having this subtype, three having a subtype of glomangioma, and one – glomangiomyoma. In some instances, these tumors can reach the parietal pleura. Two tumors were found to extend into the pleura, with ours being the third, showing that the depth of glomus tumors within the chest wall can vary. The standard treatment is surgical excision, with 12% to 33% recurrence rates, most likely resulting from incomplete removal [7]. Recurrence was reported in one patient, which happened twice. There was another patient who had been operated on previously. However, the tumor was not identified.

Most glomus tumors are benign. However, some may exhibit features suggestive of malignancy. Two distinct classifications exist: glomus tumors of uncertain malignant potential and malignant glomus tumors. A tumor is considered to have uncertain malignant potential if it has a deep location, size ≥2 cm, or mitotic activity ≥5/50 high-power field. A malignant tumor has (i) marked nuclear atypia and any level of mitotic activity or (ii) atypical mitotic figures [20]. Most analyzed cases did not report detailed pathological findings about mitotic activity or nuclear atypia. Two glomus tumors [9, 11] were classified as malignant according to criteria of deep location and size ≥2 cm. However, nowadays, without the full pathological report, it is hard to say if they would be classified as malignant. Interestingly, there were two other cases of malignant glomus tumors. In one case, a tumor was reported with metastasis in the lungs [17]. The other malignant glomus tumor was reported due to growth at an aggressive rate (size 19 cm), even though the tumor showed no malignant features with a low mitotic rate [14]. Most of the glomus tumors in the chest wall were equal to or >2 cm, and some were in deep locations so that they could be classified as glomus tumors with uncertain malignant potential.

Having a type of glomus tumor located in the chest wall can be extremely difficult to diagnose without a pathological report, as they often do not present with the classical symptoms and vary in size, depth, and location within the chest wall. Radical excision must be performed to prevent recurrence in case the tumor is malignant.
